# Supplementary material for: Are carbon emissions trading and green financial instruments synergistic? -Comprehensive quantitative research based on content analysis
Source: PLoS One. 2024 Mar 7;19(3):e0298601. doi: 10.1371/journal.pone.0298601 (PMC10919601; doi:10.1371/journal.pone.0298601)
Supplement: S1 Data — (ZIP) [file pone.0298601.s001.zip › Data/地级市Z_F工作报告.zip!/╣π╬≈/░┘╔1⁄2╩╨/└┤╫╘╣1⁄2╓┌║┼í╛┬φ┐╦╩2╛▌═°í┐_2018.docx]

一、2017年工作回顾

我们坚决贯彻落实中央、自治区和市委决策部署，牢固树立新发展理念，扎实推进供给侧结构性改革，深入开展“三年一争当”活动，以脱贫攻坚统揽经济社会发展全局，全力以赴稳增长、促改革、调结构、惠民生、防风险，经济平稳较快发展，社会和谐稳定，民族团结和睦，边疆巩固安宁，人民安居乐业。

初步统计，全年地区生产总值增长9.1%，其中第一、二、三产业增加值分别增长4.5%、10%、10%；财政收入增长8.64%；规模以上工业增加值增长10%；固定资产投资增长14.5%；社会消费品零售总额增长11.5%；外贸进出口总额增长20.3%；城镇居民人均可支配收入增长8%，农村居民人均可支配收入增长10%。

（一）全力以赴稳增长促发展，经济实现提速增效。全年地区生产总值增速继续领先全国全区平均水平，高于全区1.8个百分点，高于全国2.3个百分点。工业总产值、农业增加值、外贸进出口总额等主要指标增速排全区第一。

规模以上工业企业利润超60亿元，增长200%以上，取得前所未有的好效益。

（二）全面推进“铝二次创业”，铝产业首次突破千亿元大关。实施《关于加快铝产业“二次创业”决定》，7大煤电铝一体化项目布局基本完成，区域电网二期工程加快推进，广西华磊新材料项目、百矿田林煤电铝一体化项目竣工投产。铝产业结构趋于均衡发展，在稳定氧化铝产量的基础上，电解铝、铝材加工产量大幅增长，分别达到102万吨和314万吨。铝及配套产业产值达1162亿元，首次突破千亿元大关，进入广西千亿元产业集群行列。

（三）大力发展现代特色农业，品牌质量不断提升。持续推进芒果、油茶、柑桔、桑蚕等特色农业百万亩工程建设。新创建成各类现代特色农业（核心）示范区206个、特色养殖产业示范区（园）10个。新增农业专业合作社803家、家庭农场205家，市级重点龙头企业发展到116家。有机农产品基地认证面积、产品规模保持全区第一。新增“百色山茶油”“德保山楂”“隆林板栗”3个国家地理标志保护产品，总数达14个，保持全区第一。规模化生态化养殖水平逐步提高，获五星级生态养殖场认证17个、四星级认证76个。引进海大集团、希望集团、四川钱记等一批养殖龙头企业。“百色芒果”列入农业部种质资源圃，成为首批中国—欧盟农产品地理标志互认产品，成为“中国百强农产品区域公用品牌”。我市荣获“全国木本油料特色区域示范市”称号。第十二届世界芒果大会、中欧农产品地理标志互认工作现场会、全国有机农业基地建设经验交流会在我市召开。

（四）加快现代服务业发展，新产业新业态逐步壮大。大力推进旅游业发展，新增西林宫保府、靖西鹅泉2个国家AAAA级景区，田东荣获“全国休闲农业和乡村旅游示范县”、隆林万峰湖被评为“国家水利风景区”、靖西古龙山峡谷群被评为“广西生态旅游示范区”。星河水上乐园等一批景区建成运营。百色起义纪念公园创AAAAA工作取得新进展。全市接待旅游总人数同比增长21.79%，旅游总消费同比增长28.06%。大力发展商贸物流业，中国龙邦—越南茶岭跨境商贸物流集聚区、百色新山铝综合性服务集聚区、百煤物流园区获自治区认定。平果西南仓储冷链物流中心等一批服务业重大项目开工建设。亿丰商博城一期项目竣工使用。实施电子商务进农村工程，建成乡镇级、村级电商服务网点1260个。引进桂林银行，设立靖西长江村镇银行。市本级4家平台公司全年融资185亿元，4大基金募集资金总规模达537亿元。百色水务公司挂牌上市，填补我市上市企业的空白。健康产业布局加快，百色养生文化综合体项目、福禄河国家湿地公园、浪伏小镇等健康产业项目加快推进。大数据、云计算等新产业新业态加快形成。

（五）强化投资和项目建设，发展支撑力持续增强。开展投资环境综合整治年活动，实施项目建设攻坚战，一批重点产业、重大基础设施和民生项目落地。争取中央预算内投资项目172个、预算内投资15.6亿元。实施市层面重大项目500个、完成投资350亿元。工业和房地产投资大幅度增长，工业投资增长23.5%，房地产投资增长44.5%。左右江革命老区振兴规划、珠江—西江经济带发展规划、巴马长寿养生国际旅游区重大项目建设取得新进展。百色水库灌区可行性研究报告通过水利部审查并报国家发改委审批。百色至河池、百色至乐业、靖西至龙邦等高速路建设进展顺利，体育公园、三馆一中心、田林至西林高速公路等126个重大项目先后动工。63个中央、自治区卫生基建项目顺利开工，56个乡镇卫生院标准化项目加快推进。村级公共服务中心、乡镇污水处理项目、乡村垃圾处理项目等432个项目开工建设。全年固定资产完成1215亿元以上，投资增速14.5%以上，总量排全区第6位。

（六）扎实推进脱贫攻坚，精准脱贫取得新成效。扶贫攻坚“十大行动”和“十个到村到户”精准发力。投入财政专项扶贫资金35.4亿元，整合各类财政涉农资金26亿元。建立村级便民服务公司、村民合作社等村集体经济组织1367个，有442个贫困村集体经济收入达到2万元以上。新建、续建贫困村道路864条2496公里。实施农村危房改造1.55万户。123项易地扶贫搬迁工程全部开工，竣工住房2.18万套，4.5万人搬入新居，右江区“迎龙易地扶贫搬迁安置小镇”竣工入住。政策性金融扶贫实验示范区建设稳步实施，扶贫贷款余额达72.21亿元，整区域授信200亿元。推出“甜蜜贷”“油茶贷” “烟农贷”等金融新产品，推广“三权”抵押贷款，解决农村抵押担保困难，全市16.8万贫困户全部获得评级授信，发放扶贫小额贷款35.88亿元，覆盖贫困户7.54万户。强化深百对口扶贫协作，开工建设易地扶贫搬迁“深圳小镇”，实施人才交流、产业合作、劳务协作等项目19个，受益贫困村688个、贫困人口11万人。全国抓党建促脱贫经验交流会、全国供销社推进脱贫攻坚座谈会在我市召开，健康扶贫工程“五个一行动”在全区推广。经自治区核验，全市147个贫困村、9.99万以上贫困人口有望脱贫，右江区有望脱贫摘帽。

（七）加大生态环保力度，环境质量巩固提升。扎实开展环境保护巩固提升年活动。完成植树造林总面积63.28万亩，连续7年排全区第一，森林覆盖率达78.24%，跃居全区第一。靖西五岭森林公园获自治区批复。治理水土流失面积331平方公里，治理石漠化面积152.81平方公里。大气、水、土壤污染得到有效治理。澄碧河水库生态环境保护项目成功入选国家“水十条”项目储备库。推进铝产业、锰产业生态化建设，循环经济加快发展。华润水泥通过国家低碳产品认证，实现广西零的突破。完成中央环保督察反馈的尾矿库等问题的整改。全年环境空气质量优良率达90.1%。全面推进河长制工作。地表水水质优良率87.5%，市级饮用水水源地水质达标率保持100%。我市成功创建“国家森林城市”，是广西2017年唯一入选的城市。

（八）推进新型城镇化，城乡融合加快发展。继续推进城镇化优先发展战略，右江河谷城镇带加快建设，平果撤县设市、田阳撤县设区加快推进。百色大道右江至田阳段全线贯通，右江—田阳一体化步伐加快。迎龙片区、龙景片区城市功能更加完善，百东新区路网、公共配套设施建设加快，城市建成区违法建设专项联合治理五年行动、巩固创卫八大专项整治行动深入开展，百色城通过国家卫生城市复审验收。平果、靖西国家新型城镇化综合试点、田阳广西新型城镇化示范县建设取得新进展。德保荣获 “国家园林城市”，凌云、乐业荣获“国家卫生县城”，平果进入“中国中小城市综合实力百强县”“中国新型城镇化质量百强县”“全国中小城市投资潜力百强县”。宜居乡村建设全面启动，全区“美丽广西·宜居乡村”活动综合现场会在我市召开。百镇建设示范工程加快推进。

（九）加快门户经济发展，开放开发迈出新步伐。百色国家重点开发开放试验区、中越跨境经济合作区建设纳入国家层面推进。主动融入国家“一带一路”建设，与东盟国家在农业、教育、科技、劳务、体育、旅游等领域开展合作，取得新进展。“百色一号”专列成功对接中欧班列，开通中越跨境集装箱直通班列，融入中新互联互通国际贸易物流通道。万生隆国际商贸物流中心正式运营，龙邦边民互市区建成开园。与云南、贵州开展产能合作，百色—文山、百色—黔西南跨省（区）经济合作园区建设扎实推进。外贸扶持政策和外贸企业扶持奖励办法得到落实。全年外贸进出口总额完成166亿元，实际利用外资1.73亿美元，利用国外贷款13.6亿元。

（十）深化重点领域改革，创新发展内生动力不断增强。深化供给侧结构性改革，推进“三去一降一补”，淘汰落后铁合金产能2.22万吨，化解煤炭过剩产能9万吨，房地产去库存24.58万平方米，降成本为企业减负超10亿元。推进“放管服”改革，取消、调整行政许可事项281项。完成市、县、乡三级行政权力运行流程编制。商事制度改革释放经济活力，各类市场主体发展到16.67万户。深化国资国企改革，国资监管企业资产总额、营业收入同比分别增长67%、77%，国有资产保值增值率达112%，企业资产总额首次突破1000亿元。加快推进农村集体产权制度改革，农村土地确权登记颁证73.9万户。推进文化教育体制改革、沿边金融综合改革、农村金融改革，农村信用体系“四级联创”各项指标排全区首位，农村金融服务产品达44种。持续深化医药卫生体制改革，建立健全分级诊疗制度，全面铺开医疗联合体和医疗共同体建设。首创“高铁+公路”联运新模式，全市高铁无轨站实现全覆盖。国家高新技术产业开发区创建取得新进展，全市新增高新技术企业14家。发明专利申请量1162件，获得自治区科技计划项目立项70个。6家企业获国家标准知识产权管理体系认证，实现零的突破。千姿百色星创天地、百色学院大学生创业孵化基地分别被评为国家级“星创天地”“众创空间”。教育部在我市启动“蓝火计划”，首创西部地区产学研合作新途径。

（十一）持续改善民生福祉，群众获得感显著增强。深入推进为民办实事工程，全市民生支出完成310.88亿元，增长9.94%，占一般公共预算支出的 82.84%。51.32万城乡低保对象月人均补助水平分别从去年的300元、140元提高到330元、170元。0～3公里范围的9.85万边民每人每月获得补助130元。14万水库移民每人每年享受后期扶贫补助600元。城镇新增就业2.2万人，增长3.89%。农村劳动力转移就业新增7.02万人，增长30.1%。社会保险参保总人数达660万人次。369.28万人参加城乡居民基本医疗保险，参保率达98.27%。投资32亿元新建、改扩建教育项目1713个，一批城区、乡镇义务教育学校基础设施建成投入使用。高考一本上线人数首次突破2000人大关。实施乡村教师支持计划，全市补充中小学教师2598名。田阳、德保、凌云通过义务教育均衡发展国家级评估。乐业、平果通过自治区级评估。全市行政村全部建成“六有”标准综合服务中心。建成乡镇片区垃圾处理项目51个。改厕、改厨、改圈工程全面推进，实施农村饮水安全工程985处，解决39.8万人饮水安全问题。完成公共租赁住房和棚户区改造7927套。完成人口计生目标管理各项主要指标。启动健康百色建设。实施基层医疗卫生服务能力行动计划，开展城区医疗机构基础设施建设攻坚战，全市医疗卫生服务能力有效提高。新建村级公共服务中心318个，广播电视无线覆盖乡镇台站增加到108个。图书馆、文化馆、乡镇文化站等场所免费开放全面落实。百色壮剧《第一书记》代表广西参加全国基层文艺汇演。成功举办“丝路杯”中国—东盟乒乓球赛、第十届中国·百色国际山地户外运动挑战赛、红城百色国际半程马拉松赛等系列国际赛事，竞技体育、群众体育、体育产业与全民健身融合发展。

社会治理得到加强和创新。全市安全生产形势稳定好转，顺利通过国务院安委会、安监总局检查考评。积极创建广西食品安全示范城市，食品安全工作考核结果为A级。深化反颠覆反渗透斗争，全力开展反恐维稳工作，组织开展隐患大排查治安大整治，开展以打黑除恶为重点的“神剑”系列专项行动，建立信息化、立体化社会治安防控体系，社会治安管控能力不断提升。接边地区维稳“六联”工作经验在全区推广，维稳工作获中央表彰。国防后备力量和边防基础设施建设进一步增强，双拥共建工作取得新成果，边疆安宁稳定。

民族团结进步创建活动深入开展。宗教、侨务、保密、信访、人事人才、工商、统计、调处、应急、税务、消防、供销、水文、粮食、邮政、通信、测绘、地理信息、检验检疫、食品药品、质检、海关、海事、气象、地震、农机、国土资源、机关事务等各项工作取得新进展。地方志、档案、哲学社会科学、新闻出版、决策咨询、法律援助、妇女儿童、老龄、残疾人等事业取得新进步。工会、共青团、妇联、红十字会、文联、科协、工商联等群团组织在经济社会发展中发挥了重要作用。

全面推进依法行政，不断加强政府自身建设。把党的领导贯彻落实到依法行政全过程和各方面，各级党组织和全体党员带头尊法学法守法用法。深入开展“两学一做”学习教育制度化常态化，坚决落实中央八项规定精神和《关于新形势下党内政治生活的若干准则》《中国共产党党内监督条例》等中央重要规章条例。全面实施 “三年一争当”活动，管党治党从宽松软走向严紧实。投资环境满意度排名由2016年的全区倒数第2名，跃升至第4位。加强清廉政府建设，落实“两个责任”“一岗双责”、约谈问责制度，党风廉政建设和反腐败斗争取得新成效。出台政府规章制定办法，地方立法工作步入法制化轨道。继续推进政府法律顾问制度，行政机关负责人出庭应诉制度得到有效执行。自觉接受人大依法监督、政协民主监督和社会监督，人大代表建议和政协委员提案办结率100%。完善政府重大决策、民主决策、合法性审查的程序和机制，政府权力运行进一步规范。审计监督力度加大，绩效管理进一步加强。

二、2018年工作的总体要求和目标任务

2018年工作的总体要求是：坚持以习近平新时代中国特色社会主义思想为指导，全面贯彻落实党的十九大和中央、自治区经济工作会议精神，紧扣社会主要矛盾变化，按照高质量发展要求，统筹推进“五位一体”总体布局和协调推进“四个全面”战略布局，坚持稳中求进工作总基调，坚持新发展理念，以供给侧结构性改革为主线，以打造“三中心两区一市”为重点，攻坚突破，提质增效，全力做好稳增长、促改革、调结构、惠民生、防风险各项工作，奋力开创新时代百色经济发展新局面，为决战贫困决胜小康奠定坚实基础。

主要预期目标是：地区生产总值增长9.5%，规模以上工业增加值增长11%，财政收入增长6.5%，固定资产投资增长12%，社会消费品零售总额增长10.5%，外贸进出口总额增长20%，城镇居民人均可支配收入增长8%，农村居民人均可支配收入增长10.5%，城镇新增就业1.9万人以上，城镇登记失业率控制在4%以内。确保田阳、田东、平果、西林脱贫摘帽，贫困村和贫困人口脱贫完成自治区下达任务。节能减排控制在自治区下达的目标以内。社会各项事业不断进步，社会治理走向现代化，民生福祉持续走向新高度，人民群众幸福感明显增强。

（一）坚定不移打好脱贫攻坚战，不断提高精准脱贫质量。

强力推进产业扶贫。着力推进特色经济林、特色种养业、旅游及光伏等扶贫产业发展。统筹推进退耕还林、石漠化综合治理等生态扶贫项目。推进平果脱贫产业园建设。积极实施“五个一批”旅游脱贫工程，推动78个重点旅游贫困村规划和实施，启动20个贫困村旅游发展项目。盘活农村集体闲置资产，加快发展贫困村集体经济，力争每个贫困村都有一个主导产业。持续推进政策性金融扶贫实验示范区建设，设立产业发展基金，做大做强融资性担保体系，开发创新金融扶贫产品。

强力推进基础设施建设。推进易地扶贫搬迁，加快“深圳小镇”建设步伐，力争全市完成易地扶贫搬迁3万户10万人。继续抓好通村通屯道路新建续建、农村饮水安全工程、村级公共服务设施、农村电网改造、宽带进村等项目建设，持续改善贫困村基础设施条件。

全面整合脱贫攻坚力量。全面深化深圳百色对口扶贫协作，推动人才交流、产业合作、劳务协作、教育医疗等全方位、多领域交流协作。加大资金、技术、人才等要素向贫困村、贫困群众投入。积极对接做好与中直、区直单位定点扶贫工作。积极实施就业扶贫、健康扶贫，形成行业扶贫、专项扶贫、社会扶贫等多方力量合力攻坚的大格局。

激发贫困群众内生动力。坚持扶贫与扶志、扶智相结合，克服等靠要思想，提升贫困群众自我发展能力。开展建档立卡贫困户职业技能培训、农业技术培训，扩大就业扶贫、创业扶贫。深化教育扶贫，全面落实学生资助政策，确保每一个贫困学生都能及时得到有效帮扶。落实国家职业教育扶持政策，办好中职教育扶贫助学班，让更多贫困学生接受免费职业教育。

（二）坚定不移推进工业转型升级，大力发展先进制造业。

快推进铝二次创业。完成国家生态型铝产业示范基地产业目标，实现电解铝产能237万吨、铝加工产能320万吨，再生铝产能100万吨，铝产业工业总产值突破1500亿元。创办2018年国际铝产业高峰论坛。进一步完善区域电网，推进华磊3×35万千瓦机组、瓦村水电站发电并网，加快建设百矿德保2×35万千瓦机组。加快引黔电、滇电入百，建成百色至贵州兴义输变电线路，建成4×35万千瓦机组。坚持深化与大电网合作，提高区域电网安全保障，促进双方互利共赢。全面推进煤电铝一体化，重点推进华磊新材料40万吨、百矿苏源10万吨、百矿德保30万吨、百矿田林30万吨、百矿隆林20万吨电解铝项目全面投产达产。推进靖西天桂铝业氧化铝一期、田东百矿锦江氧化铝项目投产。发力铝精深加工和先进制造业，推进平铝集团铝别墅等铝加工产品升级。做大铝型材、铝板带箔等铝精深加工中间产品。推进平果百矿集团年产100万吨铝精深加工项目、中兴通讯用铝项目、百兴金兰铝业发动机配件等高附加值产品项目落地。推进固废综合利用、耐火材料等循环产业项目落地。培育经济发展新动能，推动互联网、大数据、人工智能和实体经济深度融合。

加强园区功能及配套建设。加快园区改革，推进市县共建工业园区，激发园区活力。推进配套产业建设，完善新山铝产业示范园、百色工业园、平果工业园、田东石化园等重点园区功能，加快建设德保、靖西、田林、隆林工业园区、右江区禄源工业园区、深圳百色产业园。推进百强碳素、强强碳素、百矿田林预焙阳极、再生铝项目建设。加快推进华银铝年产30万吨烧碱项目开工落地。加快建设园区及周边公共交通、信息网络、餐饮住宿等生活性服务设施。

推进传统工业优化升级。推动石化产业转型升级，加快田东石化总厂改革发展，稳定原油产量，支持委托加工业务增量提效，加快炼化板块退城入园。推动田东石化新城建设，发展氯碱化工、生物化工、合成材料、无水氟化铝、氟化盐等配套产业。发展碳酸钙系列产品，建好那坡龙岗30万吨铝酸钙项目。整合锰、铜、金、铁矿资源，建设百色生态型冶金产业示范基地。优化锰产业结构，发展绿色生态锰产业。加强铜产品深加工，推动精铜原料就地转化。

（三）坚定不移实施乡村振兴战略，加快推进农业农村现代化。

深入推进农村综合改革。落实中央农村工作会议精神，深化农村土地制度改革，抓好农村土地承包经营权确权登记颁证，推进农村土地“三权分置”改革。继续推进国有林场改革，集体林权制度改革，稳步推进小型水利管理体制改革，启动农业综合水价改革。深化农村集体产权制度改革，抓好农村集体资产清产核资，大力发展农村集体经济。加快农村产权流转交易平台建设，引导和推动更多的资本、技术、人才等要素向农业农村流动。加强农业农村基础工作，健全自治、法治、德治相结合的乡村治理体系。

扎实推进宜居乡村建设。紧密结合脱贫攻坚工程，巩固提升清洁乡村、生态乡村建设成果，加快建设宜居乡村。推进“产业富民、服务惠民、基础便民”三大专项活动，实施“三改六提三增”工程和“十项进村行动”，做好农村“一个中心”“六项服务”。实施世行贷款广西产业扶贫项目，与农业产业、乡村旅游、乡土文化和特色民居建设相融合，打造“一村一品”特色品牌。建设一批宜居乡村示范自然村（屯）。

做大特色优质农业。继续推进农业供给侧结构性改革，深化品种结构调整，大力开发特色主粮和特色杂粮，继续深入实施芒果、油茶、柑桔、桑蚕等百万亩工程。力争新种水果8万亩，蔬菜面积稳定在180万亩，新种桑园5万亩，茶园面积稳定在36万亩，新造改造山茶油林31万亩。全面推广现代生态养殖，争取“广西现代生态养殖场”认证率达到50%以上。做大牛羊产业，做优林下鸡产业，做稳生猪产业，做强现代生态渔业。加快建设华润五丰、钱记、华西希望等一批养殖基地。继续提升农产品品牌质量，推广果蔬、茶叶等农业标准化技术，建立覆盖农业生产全过程的标准体系及检验检测体系。深入实施化肥农药使用量零增长行动。加强农产品地理标志产品使用和保护。持续推进商标品牌战略，开展“浪伏”“壮乡河谷”等“中国驰名商标”保护。

培育新型经营主体。推进现代特色农业示范区增点、扩面、提质、升级，打造一批自治区、市、县示范区，一批乡级示范园和村级示范点。加强土地承包经营权流转服务，推进土地有序流转发展适度规模经营。引进培育农业龙头企业，加快发展农民合作社，培育一批家庭农场和专业大户。探索“农业+”等新型发展模式，发展农产品电子商务、农业休闲旅游、农业信息服务等产业，创建农村产业融合发展示范县、示范园，促进一二三产业融合发展。

做强农林产品加工业。大力发展特色农产品加工产业，推动建立右江河谷农产品加工中心，做强做优果脯加工，发展茶叶、油茶加工产品。加大桑蚕产业产学研融合力度，扩大缫丝生产，发展丝绸产业。打造百色现代林业示范区，推进林产品加工集中区建设。大力推动林板家具产业化经营，推进木材加工企业向林工贸方向发展。

（四）坚定不移壮大现代服务业，提升三产经济贡献率。

加快发展旅游业。积极发展全域旅游，深化百色与东盟各国的旅游合作，加强与云南、贵州、广东等周边地区的旅游合作，打造跨国跨省区旅游线路。推进靖西、乐业等有条件的县市创建全域旅游示范区和特色旅游名县。继续推进百色起义纪念公园创建国家AAAAA级景区。加快靖西国家旅游度假公园、凌云浩坤湖景区、右江区福禄河景区、乐业大石围“天梯”“天舟”等重大旅游项目建设。挖掘百色红色文化、民族民俗文化、古人类遗址文化等文化资源，培育旅游新项目。建设旅游集散中心、汽车营地、旅游厕所等旅游基础设施。积极发展乡村休闲旅游。

大力发展健康产业。建设区域性健康旅游养生中心。抓好自治区健康产业三年行动计划项目建设，推进鼎盛和泰养生文化园、凌云壮族茶文化休闲养生度假中心、乐业火卖户外小镇等一批健康产业项目，共建巴马长寿养生国际旅游区，促进健康与生态、旅游、养老、休闲等产业深度融合，打造健康产业集聚区。打造以森林公园、自然保护区、湿地公园为主体的森林康养旅游目的地。

大力发展商贸物流业。引进有实力的商超企业入驻迎龙、龙景、百东新区，培育新的商圈。加大电商工作力度，扩大电商覆盖面。推进中国—东盟（百色）铝产品仓储交易中心、中国—东盟商品交易中心建设。加快建设农产品专业市场，发展果蔬冷链运输，强化货源组织，壮大“百色一号”专列营运规模，抓好百色至越南的跨境班列、华东地区冷藏专列运行，力争在玉林、桂林、湛江等地新开专列上货站。

加快新产业新业态布局。利用“互联网+”技术，以实体门店、电子商务、移动互联网为载体，通过线上线下融合，大力发展共享经济、体验经济，拓展服务消费，形成新的消费增长点和发展新动能。着手在信息技术、生物技术、制造技术、新材料技术、新能源技术等领域策划一批基础性项目。在大数据、云计算、移动互联网和智能制造领域加大引进高科技企业，开展相关领域的合作研究和开发应用。

（五）坚定不移打好环境治理攻坚战，保护好百色优美生态环境。

推动绿色循环发展。加快推进百色国家气候适应型城市试点工作，开展重点适应行动，增强适应气候变化能力建设。参与全国碳排放权交易。壮大清洁能源、清洁生产、节能环保产业，培育形成绿色经济新的增长点。推进资源全面节约和循环利用，开展节能、节水、节地、节材、节矿行动，加大公共建筑节能改造。

加大生态环境保护。实施“山水林田湖草”生态保护与修复工程，继续推进退耕还林、珠江防护林、石漠化综合治理等工作。推进农村环境连片综合整治。巩固国家森林城市创建成果，实施人工造林45万亩。加强林业自然保护区、野生动植物保护区、森林公园、湿地公园、古树名木和重要水源林保护。全面落实河长制。加大耕地保护力度，严格保护永久基本农田，继续开展建设用地表土剥离再利用试点工作。实施14个水土流失综合治理工程。建设一批自治区级绿化示范村屯。

着力解决环境突出问题。组织开展第二次全国污染源普查。深入推进大气、水、土壤污染治理攻坚。同步开展右江河谷扬尘防控，强化城市道路保洁，加大执法查处力度，解决城市扬尘突出问题。划定城市高污染物禁燃区，全面淘汰不达标燃煤锅炉，推进园区企业天然气直供，抓好涉气企业超低排放改造，打好蓝天保卫战。开展澄碧湖、万峰湖治理。进一步加强下雷河整治力度。加强饮用水水源保护，保持100%达标。扎实推进土壤污染治理与修复。持续推进中央环保督察反馈意见问题整改。

全面落实环境监管责任。落实环境保护“党政同责”“一岗双责”。编制环境保护职责清单，落实部门环保职责，形成大环保工作格局。抓好环保机构监测监察执法垂直管理制度改革，加强环保基础能力建设。加强行政执法与司法联动，建立健全行业衔接机制，不断提高环境监察执法能力。

（六）坚定不移防范化解重点领域风险，保障经济稳健运行。

打好防范化解重大风险攻坚战。重点防控金融风险，促进金融与实体经济、金融与房地产、金融体系内部的良性循环。加强薄弱环节监管制度建设，坚决打击违法违规金融活动。加快筹备成立市、县地方金融监督管理局，强化风险化解、处置责任。加强政府债务风险评估、预警和监管。加强小额贷款公司管理。继续实行房地产市场分类调控，防止房价大起大落，促进房地产市场平稳健康发展。

厚植金融保障根基。加大“引金入百”力度，加强与全国性、区域性商业银行合作，吸引更多金融机构入驻百色。深化县域农村信用社改革，继续组建农村商业银行。推动设立村镇银行。加速组建百色银行。加快组建百色金融控股集团。大力引进各类保险公司在百色设立分支机构。探索发展健康保险、农业保险等专业保险。探索设立百色民间融资登记服务机构。

提升金融服务实体经济效率。创新金融产品，重点支持战略性新兴产业、企业科技创新、传统产业转型升级。合理配置信贷资源，支持重要产业、扶贫产业、重点项目建设。用好贫困地区企业上市“绿色通道”政策，加快企业上市融资。引导企业通过资产证券化等融资工具，推进企业“去杠杆”。促进创业投资基金和产业投资基金稳健发展，设立“铝二次创业引导子基金”。

（七）坚定不移推进新型城镇化，建设生态宜居城市。

推进基础设施再升级。启动建设百色大道田阳至田东至平果段。推进南昆铁路百色至威舍增二线、靖西至龙邦铁路、百色至兴义高速铁路、云南文山经百色沿边铁路等项目前期工作。建成百色至河池、靖西至龙邦高速公路。加快推进百色至乐业、田林至西林高速公路建设。推进田林至贞丰、隆林至兴义、西林至广南、乐业至望谟、田东至天峨等高速公路项目前期工作。建成凌云至凤山、乐业至凤山、田东至巴马燕洞、天生桥至隆林常么等公路。建设凌云至田林、隆林常么至西林等一批国省干道公路，推进百色龙和至德保公路等一批项目前期工作。实施县域路网攻坚工程。提级改造县乡联网公路，推进乡乡通二级（三级）路、村际联网公路建设，把农村公路建好、管好、护好、运营好。加快一批县级客运中心项目前期工作。推进客运站与“互联网+”、乡镇物流、旅客集散等功能融合发展。实施病险水库除险加固、中小河流治理工程，实施农村饮水安全巩固提升、水土保持、农田水利、小型水库、防汛抗旱项目建设。扎实推进百色水库灌区工程开工建设。

推进新型城镇化建设。加快百东新区建设。加快推进田阳撤县设区、平果撤县设市工作。修编《百色市城市总体规划（2017-2035）》《百色市历史文化名城保护规划》。启动“多规合一”，完善市区交通、绿地、停车场、燃气、地下管廊等专项规划。实施大县城战略，加快平果、靖西新型城镇化示范县、产城融合示范县建设。实现建成区面积达到159平方公里，常住人口城镇化率达到38.8%，建成区绿化覆盖率达到34.5%。加强城市生态修复、城市修补，推进海绵城市、地下综合管廊、污水处理设施建设，整治城镇黑臭水体，推进绿地系统、湿地公园建设。加强市区与高速公路连接线道路建设，完善城市道路、交通慢行系统、停车设施，开展城区道路交通秩序综合整治。加强重点镇建设，实施百镇建设示范工程，建设特色小镇、口岸小镇、民族乡，加强传统村落保护。开展国家文明城市创建。建设智慧城市，构建城市管理信息平台。出台城镇管理法规，加快成立城市综合执法机构。推动靖西 “多规合一”试点。

加快发展特色县域经济。推动各县（市、区）按主体功能区定位，差异发展，促进县域经济提质增效，综合实力提升进位，力争增加1～2个县域进入全国综合实力百强县行列，1～2个县域获得“广西科学发展先进县”，1～2个县域获得“广西科学发展进步县”。加快创建全国全域旅游示范区、广西特色旅游名县、特色农业示范区，力争把右江河谷建成“广西县域经济创新发展示范区”。稳步推进县域基础设施、公共服务设施、产业发展“三年行动计划”。完善县域经济发展组织管理、保障激励机制。鼓励发展非公有制经济，繁荣活跃县域经济。

（八）坚定不移推进改革创新，释放经济发展新活力。

继续深化投融资体制改革。继续构建农村金融“六大体系”。重点完善农村金融组织体系，力争实现村镇银行县域全覆盖。加快构建农村信用体系，开展“农村金融信用市”创建活动，构建“信用+信贷”联动模式。稳妥推进农村“两权”抵押贷款试点。推广扶贫小额信贷。扩大政策性农业保险覆盖面。推动沿边金融综合改革试验区建设，推进跨境人民币结算。支持金融机构与“一带一路”沿线国家和地区开展投融资合作。深化投融资体制改革，实施建设一批环保、水利、市政、交通、能源、信息等领域的PPP项目。统筹推进市层面重大项目500个以上，自治区层面重大项目50个以上。抓好中央预算内投资项目，争取更多专项建设基金和政府债券项目。

深化国有企业改革。稳妥推进国有企业优化重组，力争组建电力、林业、农业、金融等领域的国有企业集团。发挥社会、国有、民营等各类资本作用，加快发展混合所有制经济。做好国有困难企业解困和“僵尸企业”处置。加快剥离国有企业办社会职能，解决历史遗留问题，抓好国有企业职工家属区“三供一业”分离移交。防范化解国有企业投融资风险，确保国有资产保值增值。

继续深化“放管服”改革。继续清理规范行政许可事项、行政审批中介服务事项。完善市、县、乡三级权责清单，推动权责清单成果运用。严格执行负面清单管理。持续推进商事制度改革，实现“多证合一”“一照一码”。探索开展跨部门“双随机”联合检查，实现“双随机一公开”监管全覆盖。推进统计管理体制改革，构建现代统计治理体系。推行投资建设项目并联审批，优化审批流程。搭建大数据监管、服务、共享平台，打破“信息孤岛”，实现部门信息互联互通，变“群众跑腿”为“数据跑路”，打造24小时“不打烊”的网上政府。推进市场监管、文化、环保等领域综合执法改革。实施“红盾注册窗口服务提升工程”。加强涉企经营服务性收费监管，全面推行营改增，降低企业运营成本。加快涉企信息收集，建立企业信用约束机制。

深化住房制度改革。坚持房子是用来住的、不是用来炒的定位，大力发展住房租赁市场，加快以政府为主导的住房租赁管理服务体系建设。加强房地产市场宏观监测预警。支持商业地产引进新业态，推动房地产开发企业由单一项目开发商向城市综合运营商转变。

不断推进创新发展。建立以企业为主体、市场为导向、产学研深度融合的技术创新体系。建立铝工业、芒果产业、茶产业等技术创新载体。筹建百色铝产业研究院、博士工作站、铝产业职业技术职教中心、铝产品展示中心、铝产品检测检验中心。加快推进国家高新技术产业开发区创建工作。大力培育高新技术企业，力争申报高新技术企业5家以上。积极申报广西科技计划项目。加大高端人才引进力度，组建“蓝火计划”博士工作团百色分团。深入推进科技特派员服务机制。全面实施质量强市战略，开展质量提升行动，培育一批广西名牌、服务业品牌、优质工程、著名商标、老字号。

（九）坚定不移对外开放，创建百色国家重点开发开放试验区。

加快推进沿边开发开放。争取国家尽快设立百色国家重点开发开放试验区，力争百色边境经济合作区获得国家批准、中国龙邦—越南茶岭跨境经济合作区列入中越两国政府共同总体方案。继续做好岳圩园区详细规划，推动沿边开发开放园区基础设施建设，推进龙邦、岳圩、湖润、平孟等园区建设，配套建设岳圩进出口加工区、平孟农副产品加工项目，建成万生隆国际商贸物流中心。继续开展边境口岸基础设施大会战，完善申报受理查验等通关基础设施。推进龙邦、平孟国检试验区和龙邦智慧口岸建设，努力实现龙邦升格为国际性口岸，争取岳圩口岸恢复通关并升格为国家一类口岸。

加快推进区域（国际）合作。按照“一园两国”模式，开展铝产业等国际产能合作。与滇黔桂8市州共同推进左右江革命老区振兴规划建设，加快建设百色—文山、百色—黔西南跨省（区）经济合作园区。加强与重庆、成都等地区合作，融入渝桂新南向通道互联互通，扩大“百色一号”专列与“中欧班列”融合。

促进对外贸易加快发展。用好国家、自治区促进外贸稳定增长政策，筹措外贸发展促进资金，扶持外贸企业发展。推动边境互市贸易转型升级，促进边小贸易、边民互市繁荣发展，争取新兴互市点通过国家验收并开通运营。出台加工企业落地扶持政策，承接加工贸易产业转移，大力培育外贸经营企业，拓宽出口渠道，推动外向型企业发展国际市场。

强化招商引资。优化投资环境，巩固投资环境整治成果，提升全市投资环境满意度。加大产业招商力度，围绕铝精深加工、大健康产业、高新技术产业、战略型新兴产业等高端项目进行重点招商、精准招商。分产业、分行业组织小分队开展精准招商，积极与央企、世界企业500强、国内企业500强、民营企业500强、行业龙头企业进行对接，引进一批项目。支持沿边开发投资公司对外开展招商引资活动。继续加强与港澳台开展经贸交流与合作，推动符合条件的服务贸易投资项目在百色落地。

（十）坚定不移抓民生事业，增强人民群众幸福感。

加快发展教育事业。实施第三期学前教育行动计划、义务教育学校标准化建设、普及高中阶段教育攻坚、教育信息化等工程。引进外部资源合作办学，不断改善中小学幼儿园办学条件。建立农村留守儿童关爱保护帮扶网络，完善进城务工随迁子女就学保障机制。加快百色中心城区教育基础设施建设攻坚战。实施乡村教师支持计划。推动右江民族医学院、百色学院、百色职业学院新校区建设。建设百色新东方教育数据中心和城域网。调整优化中等职业学校布局和专业结构。统筹城乡义务教育一体化发展，年内全部县（市、区）通过城乡义务教育均衡发展国家级评估。

做好就业创业和社会保障工作。重点抓好困难群众、返乡农民工、退伍军人和大学生等群体就业，完成新增城镇就业1.9万人，农村劳动力新增转移就业4.9万人次。全面实施全民参保计划，做到应保尽保。深化人事制度和工资制度改革。加强劳动监察执法效能建设，全面治理拖欠农民工工资问题。强化对低收入群体的生活兜底保障，完善社会救助制度。实施惠民安居工程，大力推进城镇保障性安居、城市棚户区改造项目，实施棚户区改造2万套。

扎实推进健康百色建设。深入推进医药卫生体制综合改革。继续深化公立医院综合改革，加快建立现代医院管理制度，实施医保支付方式改革，扩大城乡居民大病保险。促进分级诊疗，推进医联体建设，实现50%以上的县级人民医院加入城市医联体，50%的乡镇卫生院加入县域医共体，提升家庭医生签约服务水平。严格执行药品采购“两票制”政策。继续实施基层医疗卫生服务能力行动计划，实现乡镇卫生院床位配置达标。继续推进城区医疗卫生基础设施建设攻坚战，加快市人民医院百东分院、市妇幼保健院、市疾控中心等项目建设。实施全面两孩生育政策，改善计划生育服务管理，促进基层计生基础工作规范化。创建广西食品安全示范城市。

积极发展体育事业。加大体育设施投入。办好第十届体育节、壮族三月三民族体育等系列全民健身活动。筹备和备战广西第十四届运动会，办好第五届市运动会。大力发展体育产业，继续办好“丝路杯”中国—东盟乒乓球赛、中国·百色国际山地户外运动挑战赛、全国徒步大会、红城百色国际半程马拉松赛等重大赛事。

推动文化繁荣发展。加快新建扩建市、县级文化“三馆”，加快推进市、县级妇女儿童活动中心建设，新建村级公共服务中心150个。策划创作一批大型歌舞剧、山歌剧、扶贫微电影等文化精品。加快创建历史文化名城和壮族文化生态保护区。推进文化与旅游深度融合，大力发展文化产业。

加强和创新社会治理。完善社会组织管理，推进城乡社区建设，开展农村社区建设试点创建活动。加强乡村治理法治化，积极开展社区协商，全面推行社区公共服务事项准入制度。加强社会诚信体系建设。健全应急管理体制机制，提高综合防灾减灾救灾能力。推进安全生产领域改革发展，落实安全生产责任。完善风险预防控制体系和隐患排查治理体系，深入开展重点领域隐患排查治理专项活动，坚决防范重特大事故。实施食品安全战略，创新食品药品监管方式，构建市、县、乡、村四级监管格局。继续推进“三大平安和谐区域”创建活动，深入推进法治百色、平安百色建设，构建立体化智能化社会治安防控体系。大力开展禁毒防艾等宣传教育。严厉打击黑恶势力、涉枪涉爆、毒品犯罪、电信诈骗、网络犯罪、非法集资、盗窃抢劫、传销等违法犯罪行为，扎实开展反恐防暴工作。改善各级信访基础设施，完善信访工作制度，预防化解矛盾纠纷，切实把矛盾问题化解在基层。建立健全公共法律服务体系，落实普法责任制。加强综合执法，狠抓执法规范化建设。

支持部队和国防建设。加强国防教育，加强国防动员、国防后备力量建设，继续加强边防基础设施建设和维护。深入开展双拥共建活动，促进军民融合发展。

加强和改进新时代民族工作，扎实推进民族团结进步创建活动，做好迎接自治区成立60周年大庆工作。筹备好百色起义90周年活动。扎实开展第四次全国经济普查工作。继续做好宗教、侨务、调处、统计、气象、地震、地质、测绘、水库移民、人事、编制、档案、老龄、地方志、决策咨询、哲学社会科学等工作。充分发挥工会、共青团、妇联、残联、红十字会、科协、文联、工商联等群团组织作用。

加强政府自身建设。不断增强政治本领。始终坚持全面加强党的领导，深入贯彻落实党的十九大精神，扎实推进“两学一做”学习教育常态化制度化，认真开展“不忘初心、牢记使命”主题教育，增强“四个意识”、坚定“四个自信”，在市委的坚强领导下，主动接受市人大依法监督和市政协民主监督，认真听取人大代表、政协委员及各民主党派、工商联、无党派人士、各人民团体的意见建议，办好人大代表建议和政协委员提案，讲政治、敢担当、善作为。不断增强科学发展本领。始终顺应时代潮流、把握大局大势、抢抓发展机遇，把新发展理念落实到全市工作的方方面面，多做打基础、利长远的事情，全力推动各项重点工作不断迈上新台阶。把拓宽领域、优化结构作为发展的重要方向，培育百色经济新的增长点，奋力向高质量发展。不断增强改革创新本领。始终坚持以上率下，以政府班子的具体行动带动各部门自我革新，以直面问题的勇气，啃最难啃的硬骨头、打最难打的攻坚战。创新有效办法，推行“互联网+政务服务”，提高政务效能，推动经济社会稳中有进、行稳致远。不断增强廉洁建设本领。始终坚持依法行政，坚持政府常务会集体决策制度，健全公众参与、专家论证、风险评估、合法性审查、集体讨论决定等依法决策机制，让权力在阳光下运行。坚持行政机关内部重大决策合法性审查机制，充分发挥政府法律顾问作用，严格行政决策过错责任追究。坚持清正廉洁，带头遵守法纪和政治规矩，坚决落实中央八项规定精神，时刻把纪律挺在前面，打造廉洁政府。不断增强狠抓落实本领。始终以永不懈怠的精神状态和一往无前的奋斗姿态，以雷厉风行、真抓实干、马上就办的工作作风，推进重大部署、重大决策、重大工程和重大项目的落实，以钉钉子精神做实、做细、做好各项工作，让决策落地生根，让蓝图变成现实。
